# Gender-specific factors associated with the use of mental health services for suicidal ideation: Results from the 2013 Korean Community Health Survey

**DOI:** 10.1371/journal.pone.0189799

**Published:** 2017-12-18

**Authors:** Mina Kim, Young-Hoon Lee

**Affiliations:** 1 Department of Nursing, Graduate School, Chonnam National University, Dong-gu, Gwangju, Republic of Korea; 2 Department of Counseling Psychology, Graduate School of Dongshin University, Naju, Jeonnam, Republic of Korea; 3 Department of Preventive Medicine & Institute of Wonkwang Medical Science, Wonkwang University School of Medicine, Iksan, Jeonbuk, Republic of Korea; 4 Regional Cardiocerebrovascular Center, Wonkwang University Hospital, Iksan, Jeonbuk, Republic of Korea; Stellenbosch University, SOUTH AFRICA

## Abstract

This study examined gender-specific factors associated with the use of mental health services (MHS) for suicidal ideation (SI). We included data on 6,768 males and 12,475 females who had experienced SI over the past year from the nationwide 2013 Korean Community Health Survey. These individuals were grouped as MHS users for SI if they had received professional counseling at medical institutions, professional counseling agencies, or community health centers for SI-related problems. Their information on sociodemographic factors, socio-familial relationships, health behaviors, and health status were included as exposures in a logistic regression analysis. Of the 19,243 individuals, 7.0% of the males and 10.5% of the females used MHS for SI treatment. For males with SI, living in an urban area, being a widower, and having unhealthy behaviors (frequent alcohol consumption and infrequent walking) were associated with underuse of MHS. For females with SI, frequent contact with friends, low level of religious activity, and good self-rated health were associated with underuse of MHS. For both males and females, those who were younger, completed higher education, and experienced depression/suicide attempts in the past year were more likely to use MHS for SI. These findings suggest that gender-specific factors should be used to inform suicide prevention strategies.

## Introduction

Suicide is one of the most important public health issues in the world. Suicide rates vary widely across countries, and Korea has twice as high of a suicide rate compared with other Organization for Economic Cooperation and Development countries [1]. Korea’s suicide rate was 26.5 deaths per 100,000 people and was the fifth leading cause of death in 2015 [1]. Although most people with suicidal ideation (SI) do not die by suicide, SI is closely related to suicide attempts, and these suicidal behaviors are positively related to death by suicide [2]. Therefore, linking people with SI to appropriate mental health services (MHS) is an important strategy for preventing suicide. Studies have found that the majority of people at high risk for suicide do not use any form of MHS [3].

Suicide-related characteristics differ depending on gender. In almost all countries, males have a higher suicide rate than that of females [4], but females attempt suicide more often than do males [5]. Gender-specific associated factors should be considered in the prevention and management of suicide attempts, and the gender-specific factors underlying the use of MHS for SI should also be investigated. Previous studies have examined the associations between socioeconomic factors and use of MHS for SI [3,6–8]. However, there is a lack of research on the differences in factors related to the use of MHS by gender [9]. A Korean study reported that only 8.2% of adults with SI have used MHS for their mental health problems, and revealed the socioeconomic factors related to the use of MHS in the population with SI from the National Health Survey [10]. However, the small sample, limited independent variables, and the lack of an analysis by gender prevented a comprehensive understanding of the use of MHS among the general population with SI.

Therefore, the purpose of this study was to provide information for establishing improved and customized suicide prevention policies by identifying gender-specific factors associated with the use of MHS for SI and by examining gender-specific barriers for those seeking help from MHS.

## Methods

### Study population

The Korean Community Health Survey (KCHS) conducted by the Korea Centers for Disease Control and Prevention is a nationwide survey that has been carried out annually since 2008 by trained surveyors using a computer-assisted personal interviewing method. Multistage, stratified, and random sampling was used to select representative households in 253 local Korean communities based on resident registration information resulting from surveying an average of 900 individuals from each local community. This study used data from the 2013 KCHS collected from August 16, 2013 to October 31, 2013. A total of 228,781 individuals (102,722 males and 106,059 females) aged ≥ 19 years participated in the 2013 survey. The 2013 KCHS provides population-based estimates of health indicators, including health status, morbidity, health service use, and health behaviors using a standardized questionnaire consisting of 258 questions. After excluding participants with missing data regarding sociodemographic variables, socio-familial relationships, health behaviors, and health status, 19,243 subjects (6,768 males and 12,475 females) who had experienced SI over the past year were included in the final analysis. This study was conducted in accordance with the Declaration of Helsinki guidelines. Written informed consent was obtained from all participants in the KCHS. The study protocol was approved by the Institutional Review Board of Wonkwang University Hospital (WKUH 2017-05-018).

### Outcome and variables measurements

Suicide-related behaviors, including SI, suicide attempts, and use of MHS for SI, were evaluated using a questionnaire. SI was defined as having had thoughts of wanting to die in the past year. The use of MHS for SI included subjects who had received professional counseling at a medical institution, professional counseling agency, or community health center for SI-related problems in the past year.

Information on each subject’s sociodemographic factors, socio-familial relationships, health behaviors, and health status was collected using a questionnaire. A detailed description of the variables used in this study is provided in Table 1.

**Table 1:** Independent variables used in this study.

Variable	Question	Category
**Sociodemographic factors**		
Gender	What is your gender?	Male or female
Age group	What is your age?	19–44, 45–64, 64–74, or ≥75 years
Residence type	Where is your residence?	Urban or rural
Marital status	Have you ever been married (including a de facto marriage)? Which of the following is your current marital status? (1) I have a spouse and we live together (2) I have a spouse but we do not live together (3) No spouse due to death (4) No spouse due to divorce	Never married, married, divorced/separated, or widowed
Household composition	Which of the following is your household type?	Living alone, single generation, two generations, or three generations
Education level	Where did you go to school? Did you graduate from school?	No formal education, primary school, middle or high school, or college and higher
Monthly household income	What was your average monthly household income in the past year, including wages, real estate income, pensions, interest, government subsidies, and allowances for relatives or children?	≤1, 1.01–2, 2.01–4, or ≥4.01 million won
Employment status	What is your occupation? Please list the specific type of work.	Employed, unemployed, or housewife/student
National Basic Livelihood Security status	Does your household currently receive National Basic Livelihood Security?	Recipient or non-recipient
**Socio-familial relationships**		
Contact with family	How often do you see or contact your closest relatives (including your family)?	<1 time or ≥1 time per month
Contact with neighbors	How often do you see or contact your closest neighbors?	<1 time or ≥1 time per month
Contact with friends	How often do you see or communicate with your closest friends (except your neighbors)?	<1 time or ≥1 time per month
Religious activities	Do you participate in religious activities once per month or more?	<1 time or ≥1 time per month
Activities with friends	Do you regularly participate in activities with friends once per month or more?	<1 time or ≥1 time per month
Leisure activities	Do you participate regularly in leisure activities at least once per month?	<1 time or ≥1 time per month
Charitable activities	Do you participate in charitable activities once per month or more?	<1 time or ≥1 time per month
**Health behaviors and health status**		
Smoking status	Have you smoked more than 5 packs (100 cigarettes) during your life? Do you smoke now? (1) I smoke every day (2) Sometimes I smoke (3) I smoked in the past but I do not smoke now	Never, former, or current smoker
Frequency of alcohol use	Have you ever drunk more than one drink in your life? Have you been drinking for the last year? How often do you drink alcohol?	None, ≤1, 2–3, or ≥4 times per week
Walking activity	How many days did you walk for at least 10 minutes at a time in the last week?	≤2 or ≥3 times per week
Sleep duration	How many hours a day do you usually sleep?	≤6, 7–8, or ≥9 h per day
Self-rated health	What do you think about your health?	Good, fair, or poor
Perceived daily stress	How often do you feel stressed in your daily life?	Low or high
Experience of depressed mood	Have you ever felt sad or desperate for more than two consecutive months during the past year to the extent that it interferes with your daily life?	No or yes
Number of chronic diseases	Have you been diagnosed with any of the following diseases: obesity, hypertension, diabetes, dyslipidemia, stroke, myocardial infarction, angina pectoris, osteoarthritis, osteoporosis, asthma, or hepatitis B?	≤2 and ≥3
Diagnosis of depression	Have you been diagnosed with depression?	No or yes
Suicide attempts	Have you attempted suicide in the last year?	No or yes

### Statistical analysis

The participants’ characteristics were compared according to gender using the chi-square test. After adjusting for all of the evaluated covariates, the adjusted odds ratio (aOR) with 95% confidence interval (CI) of using MHS for SI were subjected to multivariate logistic regression analysis. Logistic regression analyses were performed separately for males and females. All statistical analyses were performed using SPSS Statistics for Windows ver. 22.0 (IBM Co., Armonk, NY, USA). A *P*-value < 0.05 was considered significant.

## Results

### Sample characteristics by gender

Characteristics according to gender are presented in Table 2. Of the 19,243 subjects who had experienced SI, 1,780 (9.3%) received professional counseling for SI, which was a significantly higher proportion in females (10.5%) than in males (7.0%). There were significant differences in age group, residence type, marital status, household composition, education level, monthly household income, and employment status between the genders; no significant difference in National Basic Livelihood Security (NBLS) status according to gender was observed. Compared with males, females contacted their family and neighbors more frequently and participated in religious activities more often. In contrast, males contacted their friends more frequently and participated more often in activities with friends and leisure than females. A greater proportion of males than females were current or past smokers and males tended to drink more frequently, but walk less frequently. Sleep duration and self-rated health status differed significantly according to gender. Compared with males, the proportions with high perceived daily stress, experienced depressive mood, having more than three chronic diseases, and diagnosis of depression were higher in females. The proportion of suicide attempts was higher in males (4.6%) than in females (3.9%).

**Table 2:** Comparison of variables according to gender.

Variable	Males [n = 6,768]	Females [n = 12,475]	*P*
**Dependent variable**			
Mental health service for suicidal ideation			<0.001
Non-use	6,297 (93.0)	11,166 (89.5)	
Use	471 (7.0)	1,309 (10.5)	
**Sociodemographic factors**			
Age group			0.019
19–44 years	1,714 (25.3)	3,303 (26.5)	
45–64 years	2,541 (37.5)	4,775 (38.3)	
65–74 years	1,487 (22.0)	2,513 (20.1)	
≥75 years	1,026 (15.2)	1,884 (15.1)	
Residence type			<0.001
Urban	3,581 (52.9)	7,059 (56.6)	
Rural	3,187 (47.1)	5,416 (43.4)	
Marital status			<0.001
Married	4,510 (66.6)	7,380 (59.2)	
Never married	1,020 (15.1)	1,118 (9.0)	
Divorced/separated	773 (11.4)	1,075 (8.6)	
Widowed	465 (6.9)	2,902 (23.3)	
Household composition			<0.001
Living alone	1,049 (15.5)	2,248 (18.0)	
Single generation	2,301 (34.0)	3,377 (27.1)	
Two generations	2,901 (42.9)	5,597 (44.9)	
Three generations	517 (7.6)	1,253 (10.0)	
Education level			<0.001
No formal education	334 (4.9)	1,902 (15.2)	
Primary school	1,544 (22.8)	3,431 (27.5)	
Middle or high school	3,201 (47.3)	4,842 (38.8)	
College or higher	1,689 (25.0)	2,300 (18.4)	
Monthly household income			0.034
≤1 million won	2,646 (39.1)	4,768 (38.2)	
1.01–2 million won	1,473 (21.8)	2,595 (20.8)	
2.01–4 million won	1,652 (24.4)	3,099 (24.8)	
4.01 million won	997 (14.7)	2,013 (16.1)	
Employment status			<0.001
Employed	4,181 (61.8)	5,659 (45.4)	
Unemployed	2,462 (36.4)	1,571 (12.6)	
Housewife or student	125 (1.8)	5,245 (42.0)	
National Basic Livelihood Security			0.113
Non-recipient	6,191 (91.5)	11,493 (92.1)	
Recipient	577 (8.5)	982 (7.9)	
**Socio-familial relationships**			
Contact with family			<0.001
<1 time per month	1,821 (26.9)	2,441 (19.6)	
≥1 time per month	4,947 (73.1)	10,034 (80.4)	
Contact with neighbors			<0.001
<1 time per month	2,302 (34.0)	3,446 (27.6)	
≥1 time per month	4,466 (66.0)	9,029 (72.4)	
Contact with friends			0.006
<1 time per month	2,081 (30.7)	4,077 (32.7)	
≥1 time per month	4,687 (69.3)	8,398 (67.3)	
Religious activities			<0.001
<1 time per month	5,492 (81.1)	8,340 (66.9)	
≥1 time per month	1,276 (18.9)	4,135 (33.1)	
Activities with friends			0.007
<1 time per month	3,641 (53.8)	6,962 (55.8)	
≥1 time per month	3,127 (46.2)	5,513 (44.2)	
Leisure activities			<0.001
<1 time per month	5,374 (79.4)	10,676 (85.6)	
≥1 time per month	1,394 (20.6)	1,799 (14.4)	
Charitable activities			0.893
<1 time per month	6,413 (94.8)	11,815 (94.7)	
≥1 time per month	355 (5.2)	660 (5.3)	
**Health behaviors and health status**			
Smoking status			<0.001
Never smokers	1,149 (17.0)	11,113 (89.1)	
Former smokers	2,472 (36.5)	533 (4.3)	
Current smokers	3,147 (46.5)	829 (6.6)	
Frequency of alcohol use			<0.001
None	1,867 (27.6)	5,623 (45.1)	
≤1 time per week	2,266 (33.5)	5,550 (44.5)	
2–3 times per week	1,266 (18.7)	946 (7.6)	
≥4 times per week	1,369 (20.2)	356 (2.9)	
Walking activity			0.002
≤2 times per week	2,834 (41.9)	4,939 (39.6)	
≥3 times per week	3,934 (58.1)	7,536 (60.4)	
Sleep duration			<0.001
≤6 h per day	3,493 (51.6)	6,990 (56.0)	
7–8 h	2,818 (41.6)	4,934 (39.6)	
≥9 h per day	457 (6.8)	551 (4.4)	
Self-rated health			<0.001
Poor	2,884 (42.6)	5,662 (45.4)	
Fair	2,406 (35.5)	4,703 (37.7)	
Good	1,478 (21.8)	2,110 (16.9)	
Perceived daily stress			<0.001
Low	3,001 (44.3)	4,955 (39.7)	
High	3,767 (55.7)	7,520 (60.3)	
Experience of depressed mood			<0.001
No	4,799 (70.9)	8,146 (65.3)	
Yes	1,969 (29.1)	4,329 (34.7)	
Number of chronic diseases			<0.001
≤2	5,653 (83.5)	9,233 (74.0)	
≥3	1,115 (16.5)	3,242 (26.0)	
Diagnosis of depression			<0.001
No	6,171 (91.2)	10,657 (85.4)	
Yes	597 (8.8)	1,818 (14.6)	
Suicide attempt(s)			0.009
No	6,454 (95.4)	11,994 (96.1)	
Yes	314 (4.6)	481 (3.9)	

### Gender-specific factors predicting the use of MHS for SI

Univariate analyses showed that among males, sociodemographic factors (age group, residence type, marital status, household composition, education level, employment status, and NBLS), socio-familial relationships (contact with family, contact with neighbors, religious activity, and activities with friends), and health behaviors and health status (smoking status, frequency of alcohol use, walking activity, self-rated health, perceived daily stress, experience of depressed mood, number of chronic diseases, diagnosis of depression, and suicide attempts) were significantly associated with the use of MHS for SI. Among females, sociodemographic factors (age group, residence type, marital status, education level, employment status, and NBLS), socio-familial relationships (contact with family, contact with neighbors, religious activity, activities with friends, leisure activities, and charitable activities), and health behaviors and health status (smoking status, walking activity, sleep duration, self-rated health, perceived daily stress, experience of depressed mood, diagnosis of depression, and suicide attempts) were significantly associated with the use of MHS for SI (data not shown).

Fully adjusted gender-specific relationships between the use of MHS for SI and sociodemographic factors, socio-familial relationships, and health behaviors and health status, as determined by logistic regression analysis, are presented in Tables 3 (males) and 4 (females). After full adjustment, the decreasing trend in the ORs for the use of MHS with increasing age remained significant for both genders. Compared with those aged 19–44 years, the aORs (95% CI) for use of MHS among those aged 45–64, 65–74, and ≥75 years were 0.59 (0.41–0.84), 0.54 (0.34–0.88), and 0.36 (0.19–0.67), respectively, for males and 0.85 (0.69–1.07), 0.67 (0.50–0.90), and 0.56 (0.38–0.82), respectively, for females. Males living in urban areas had a lower OR for using MHS than did males living in rural areas (aOR = 0.77, 95% CI = 0.59–0.99). After adjusting for related variables, the significant associations between marital status and use of MHS in the unadjusted model were no longer significant. Meanwhile, in the fully adjusted model, compared with married persons, use of MHS was significantly higher in widowers (aOR = 0.44, 95% CI = 0.23–0.88) but not in widows. The least-educated subjects (no formal education) had a lower OR for using MHS among both males (aOR = 0.39, 95% CI = 0.16–0.91) and females (aOR = 0.48, 95% CI = 0.33–0.70) compared with the most-educated subjects (college or higher). No significant association between employment status, NBLS and use of MHS was observed in either gender after adjustment for related factors.

**Table 3:** Factors predictive of the use of mental health services for suicidal ideation by logistic regression analysis among males.

Variables	Fully adjusted OR (95% CI)	*P*
**Sociodemographic factors**		
Age group		
19–44 years	1.00	
45–64 years	0.59 (0.41–0.84)	0.004
65–74 years	0.54 (0.34–0.88)	0.013
≥75 years	0.36 (0.19–0.67)	0.001
Residence type		
Rural	1.00	
Urban	0.77 (0.59–0.99)	0.048
Marital status		
Married	1.00	
Never married	1.24 (0.85–1.83)	0.268
Divorced/separated	1.27 (0.83–1.93)	0.271
Widowed	0.44 (0.23–0.88)	0.019
Household composition		
Living alone	0.80 (0.53–1.20)	0.273
Single generation	0.83 (0.59–1.16)	0.268
Two generations	1.00	
Three generations	0.95 (0.59–1.55)	0.841
Education level		
College or higher	1.00	
Middle or high school	0.73 (0.54–0.98)	0.036
Primary school	0.74 (0.49–1.13)	0.163
No formal education	0.39 (0.16–0.91)	0.030
Employment status		
Housewife or student	1.00	
Employed	0.82 (0.40–1.68)	0.582
Unemployed	0.99 (0.47–2.09)	0.980
National Basic Livelihood Security		
Non-recipient	1.00	
Recipient	1.30 (0.89–1.92)	0.177
**Socio-familial relationships**		
Contact with family		
<1 time per month	1.00	
≥1 time per month	1.18 (0.90–1.55)	0.228
Contact with neighbors		
<1 time per month	1.00	
≥1 time per month	1.17 (0.88–1.54)	0.280
Religious activity		
<1 time per month	1.00	
≥1 time per month	1.17 (0.88–1.56)	0.281
Activities with friends		
<1 time per month	1.00	
≥1 time per month	1.07 (0.83–1.39)	0.583
**Health behaviors and health status**		
Smoking status		
Never smokers	1.00	
Former smokers	0.85 (0.60–1.21)	0.367
Current smokers	0.88 (0.63–1.22)	0.430
Frequency of alcohol use		
None	1.00	
≤1 time per week	0.97 (0.71–1.33)	0.850
2–3 times per week	0.77 (0.52–1.16)	0.212
≥4 times per week	0.58 (0.39–0.86)	0.007
Walking activity		
≥3 times per week	1.00	
≤2 times per week	0.70 (0.55–0.90)	0.005
Self-rated health		
Poor	1.00	
Fair	0.78 (0.57–1.05)	0.102
Good	0.77 (0.54–1.11)	0.165
Perceived daily stress		
Low	1.00	
High	1.25 (0.96–1.63)	0.098
Experience of depressed mood		
No	1.00	
Yes	2.18 (1.70–2.79)	<0.001
Number of chronic diseases		
≤2	1.00	
≥3	1.15 (0.83–1.58)	0.403
Diagnosis of depression		
No	1.00	
Yes	29.95 (23.38–38.36)	<0.001
Suicide attempts		
No	1.00	
Yes	2.31 (1.58–3.39)	<0.001
OR, odds ratio; CI, confidence interval		

**Table 4:** Factors predicting of the use of mental health services for suicidal ideation by logistic regression analysis among females.

Variables	Fully adjusted OR (95% CI)	*P*
**Sociodemographic factors**		
Age group		
19–44 years	1.00	
45–64 years	0.85 (0.69–1.07)	0.150
65–74 years	0.67 (0.50–0.90)	0.007
≥75 years	0.56 (0.38–0.82)	0.003
Residence type		
Rural	1.00	
Urban	0.98 (0.83–1.15)	0.793
Marital status		
Married	1.00	
Never married	1.28 (0.96–1.70)	0.094
Divorced/separated	0.91 (0.70–1.17)	0.455
Widowed	0.85 (0.68–1.07)	0.160
Education level		
College or higher	1.00	
Middle or high school	0.91 (0.73–1.14)	0.426
Primary school	0.69 (0.52–0.92)	0.012
No formal education	0.48 (0.33–0.70)	<0.001
Employment status		
Housewife or student	1.00	
Employed	0.85 (0.72–1.01)	0.061
Unemployed	1.05 (0.82–1.36)	0.692
National Basic Livelihood Security		
Non-recipient	1.00	
Recipient	0.97 (0.75–1.26)	0.828
**Socio-familial relationships**		
Contact with family		
<1 time per month	1.00	
≥1 time per month	0.94 (0.78–1.14)	0.544
Contact with neighbors		
<1 time per month	1.00	
≥1 time per month	0.93 (0.78–1.11)	0.427
Contact with friends		
<1 time per month	1.00	
≥1 time per month	0.80 (0.68–0.95)	0.011
Religious activity		
<1 time per month	1.00	
≥1 time per month	1.28 (1.09–1.49)	0.002
Activities with friends		
<1 time per month	1.00	
≥1 time per month	1.01 (0.86–1.19)	0.910
Leisure activities		
<1 time per month	1.00	
≥1 time per month	1.19 (0.96–1.47)	0.112
Charitable activities		
<1 time per month	1.00	
≥1 time per month	1.31 (0.96–1.78)	0.087
**Health behaviors and health status**		
Smoking status		
Never smokers	1.00	
Former smokers	0.86 (0.60–1.23)	0.406
Current smokers	0.88 (0.67–1.15)	0.343
Walking activity		
≥3 times per week	1.00	
≤2 times per week	0.92 (0.79–1.07)	0.284
Sleep duration		
≤6 h per day	1.07 (0.92–1.25)	0.372
7–8 h	1.00	
≥9 h per day	1.04 (0.74–1.48)	0.822
Self-rated health		
Poor	1.00	
Fair	0.98 (0.82–1.17)	0.829
Good	0.78 (0.61–0.99)	0.043
Perceived daily stress		
Low	1.00	
High	1.03 (0.88–1.22)	0.697
Experience of depressed mood		
No	1.00	
Yes	2.15 (1.85–2.50)	<0.001
Diagnosis of depression		
No	1.00	
Yes	27.43 (23.55–31.95)	<0.001
Suicide attempts		
No	1.00	
Yes	3.12 (2.40–4.06)	<0.001
OR, odds ratio; CI, confidence interval		

After adjusting for related variables, more religious activity (aOR = 1.28, 95% CI = 1.09–1.49) was positively associated, and more contact with friends (aOR = 0.80, 95% CI = 0.68–0.95) was negatively associated with the use of MHS in females. However, none of the socio-familial factors showed an association in males after full adjustment. Compared with non-drinkers, the OR of using MHS was lower in males who drank ≥ 4 times/week in the fully adjusted model (aOR = 0.58, 95% CI = 0.39–0.86). Although walking activity was negatively associated with the use of MHS in both genders in the unadjusted model, only males who walked less had a lower likelihood of using MHS after full adjustment (aOR = 0.70, 95% CI = 0.55–0.90). After adjusting for related factors, the OR for using MHS was greater in females with good health (aOR = 0.78, 95% CI = 0.61–0.99) than in those with poor health. After adjusting for related factors, the OR for using MHS among those who experienced depressed mood was significantly higher (2.18-fold, 95% CI = 1.70–2.79) in males and (2.15-fold, CI = 1.85–2.50) higher in females compared with those who did not experience depressed mood. The OR for using MHS was significantly higher among males (aOR = 29.95, 95% CI = 23.38–38.36) and females (aOR = 27.43, 95% CI = 23.55–31.95) who were diagnosed with depression compared with those who were not diagnosed with depression. Males (aOR = 2.31, 95% CI = 1.58–3.39) and females (aOR = 3.12, 95% CI = 2.40–4.06) who had attempted suicide had a higher OR for using MHS compared with those who had not attempted suicide.

## Discussion

Among the community-dwelling general population with SI, this study examined gender-specific associations between sociodemographic factors, socio-familial relationships, health behaviors, and health status with use of MHS. Significant relationships were observed between using MHS and residence type, marital status, frequency of alcohol use, and walking activity in males, whereas contact with friends, religious activity, and self-rated health were significantly associated with use of MHS in females. In contrast, our findings showed that age, education level, experience of depressed mood, depression diagnosis, and suicide attempts were associated with the use of MHS for SI in males and females.

In a review of 12 studies, the utilization rate of professional mental health providers by people with SI, suicide plans, and/or suicide attempts during the past year was approximately 29.5% [3]. We defined the use of MHS as professional counseling at a medical institution, professional counseling agency, or community health center for SI-related problems. Especially, in this study, the use of mental health counseling only included counseling received from visiting a mental health professional and did not include telephone or internet counseling. In addition, only SI-related issues, and no other mental health problems, were included in the definition of the use of MHS for SI. In this study, 7.0% of males and 10.5% of females (9.3% in total) used MHS for SI, which is slightly higher than the rate reported in a previous Korean study [10].

Previous studies have consistently reported that age is an important predictor of using MHS for SI [8,10]. Older people are less sensitive to psychiatric symptoms, whereas younger people are more aware of the need for MHS [11,12]. Similar to previous studies, the present study identified that age and use of MHS for SI were inversely associated in males and females, although the magnitude of the association was greater in males. Education level, which is an important indicator of socioeconomic status, was positively associated with the use of MHS for SI in both genders in the present study. A previous study reported that education level was not a significant determinant of utilizing MHS in males or females [9], but other studies have shown a significant association between education level and the use of MHS for SI [8,10,13]. Highly educated people generally use MHS because they are less stigmatized about mental illness and have a positive attitude toward the effectiveness of treatment for mental illness [14,15], whereas those with lower levels of education are more economically burdened by MHS and are less aware of mental illness problems and treatment, resulting in limited use of MHS [15,16]. In addition, older age and lower education levels are related to less knowledge about suicide, which may affect seeking help for SI [17].

Marital status, especially widowed or divorced, has a greater influence on suicide mortality in males than females [18,19]. In addition, our previous study identified that widowed, divorced, or separated males attempted suicide significantly more frequently than did married males, but widowed, divorced, or separated females attempted suicide significantly less frequently than did married females [20]. In the present study, the frequency of MHS use was significantly lower in widowers compared with married persons, indicating that widowers are vulnerable to suicide in Korea. Death or divorce of a spouse is a significant risk factor for suicide in both genders, but the impact on females is somewhat weaker, because females continue to receive support through social and family connections even after losing their spouse [21]. In addition, traditional male gender roles, including greater levels of strength and independence, often prevent them from seeking help for suicidal feelings and depression [22]. A Korean study reported that the risk of not using MHS was 2.75-fold higher for widows than for married people, but the results were not evaluated by gender [10]. Two Canadian studies in adults with SI reported an association between marital status and use of MHS [8,9]. One study found a significantly higher use of MHS in unmarried and divorced people than in married people [8], while the other study reported that marital status and use of MHS were not related in males or females [9].

Social interaction and religious involvement are independently related to suicide [23]. One study showed that the incidence of suicide decreases with increasing social integration, indicating that higher levels of social integration are associated with protection against suicide in females [24]. In our study, less contact with friends and greater religious activities were significantly related to the use of MHS for SI in females only. These results are difficult to explain. However, determining the extent of involvement in a range of social relationships may provide useful information for assessing suicidal risk and establishing a tailored strategy [24]. It is possible that females with a better social support system may seek help from people around them rather than seeking help through MHS, while males do not seek such help. Further research is needed on the gender-specific associations between socio-familial relationships and the use of MHS.

In this study, unhealthy behaviors, such as frequent drinking and lack of exercise, were significantly associated with not using MHS for SI in males, but not in females. Although not significant on multivariate analysis, the univariate analysis showed a significant association between current smoking and MHS use in males. To the best of our knowledge, no previous studies have examined gender-specific associations between health-related behaviors and MHS utilization by adults with SI. Unlike females, males who engage in unhealthy behaviors are more likely not to use MHS; thus, preventing suicide among males may require healthcare policies and societal concern to encourage the use of MHS for SI in males who are behaving in an unhealthy manner. Depression is an important risk factor for suicide [25]. Previous studies have shown that depression, psychiatric disorders, and psychiatric distress are important underlying factors for MHS use [8–10,26]. Our study found that a diagnosis of depression, experience of depressed mood, and suicide attempts were independently associated with the use of MHS in both genders, of which depression was the most potent factor in the use of MHS.

Some limitations should be considered when interpreting the results of this study. First, due to the cross-sectional design, this study could not derive causal relationships. Second, information on SI, suicide attempts, and the use of MHS was collected retrospectively, so recall bias may have occurred. Third, although socio-familial relationships were included in our analysis, the distribution of community resources such as medical institutions and community health centers, access to healthcare facilities, and regional cultural differences that affect the use of MHS were not included [13]. Fourth, attitudes and stigma about mental illness or MHS use were not evaluated. Promoting a positive attitude and reducing the stigma associated with the use of MHS in the general public may facilitate seeking the help of mental health professionals. Lastly, although this study included a large number of samples, statistical significance can be influenced by sample size and the variance of variables between the genders. Despite these limitations, in this study, we analyzed data from a national health survey and assessed a representative large-scale general population. In addition, multiple covariates, such as socioeconomic information, socio-familial relationships, health behaviors, and health status, were investigated simultaneously according to gender.

## Conclusions

This study identified gender-specific factors associated with the use of MHS in the general Korean population with SI. These findings suggest that gender-specific factors should be used to inform suicide prevention strategies. Further studies are required to demonstrate gender-specific causal relationships between MHS utilization and related factors in individuals with SI.
